# Phenolic Glycoside Monomer from *Reed Rhizome* Inhibits Melanin Production *via* PI3K-Akt and Ras-Raf-MEK-ERK Pathways

**DOI:** 10.2174/0109298673341645240919072455

**Published:** 2024-09-27

**Authors:** Meijun Pang, Hong Yao, Kechen Bao, Ruitian Xu, Rongjiao Xi, Rui Peng, Hui Zhi, Kuo Zhang, Runnan He, Yunfei Du, Yanfang Su, Xiuyun Liu, Dong Ming

**Affiliations:** 1 Medical School, Tianjin University, 92 Weijin Road, Nankai District, Tianjin, 300072, China;; 2 Haihe Laboratory of Brain-Computer Interaction and Human-Machine Integration, Tianjin, 300072, China;; 3 State Key Laboratory of Advanced Medical Materials and Devices, Tianjin, 300072, China;; 4 School of Pharmaceutical Science and Technology, Tianjin University, 92 Weijin Road, Nankai District, 300072, Tianjin, China

**Keywords:** *Reed Rhizome* extract, anti-melanogenesis, zebrafish model, tyrosinase, age spots, radicals

## Abstract

**Introduction:**

Melanogenesis, the process responsible for melanin production, is a critical determinant of skin pigmentation. Dysregulation of this process can lead to hyperpigmentation disorders.

**Methods:**

In this study, we identified a novel *Reed Rhizome* extract, (1'S, 2'S)-syringyl glycerol 3'-O-β-D-glucopyranoside (compound **5**), and evaluated its anti-melanogenic potential in zebrafish models and *in vitro* assays. Compound **5** inhibited melanin synthesis by 36.66% ± 14.00% and tyrosinase *in vivo* by 48.26% ± 6.94%, surpassing the inhibitory effects of arbutin. Network pharmacological analysis revealed key targets, including HSP90AA1, HRAS, and PIK3R1, potentially involved in the anti-melanogenic effects of compound **5**.

**Results:**

Molecular docking studies supported the interactions between compound **5** and these targets. Further, gene expression analysis in zebrafish indicated that compound **5** up-regulates *hsp90aa1.1*, *hrasa*, and *pik3r1*, and subsequently down-regulating *mitfa*, *tyr*, and *tyrp1*, critical genes in melanogenesis.

**Conclusion:**

These findings suggest that compound **5** inhibits melanin production *via* PI3K-Akt and Ras-Raf-MEK-ERK signaling pathways, positioning it as a promising candidate for the treatment of hyperpigmentation.

## 1 INTRODUCTION

Melanin is an intracellular particle produced by melanocytes and delivered to keratinocytes, which has the function of absorbing ultraviolet rays, antioxidant and scavenging free radicals [1]. However, the aberrant accumulation of melanin can lead to various dermatological disorders, including melasma, age spots, and other hyperpigmentation conditions. Consequently, there is a growing interest in identifying novel compounds that can effectively modulate melanin synthesis.

Arbutin, a well-known tyrosinase inhibitor, has been widely used as a skin-lightening agent in cosmetic products. Despite its popularity, the quest for more potent and safer melanogenesis inhibitors continues, as the efficacy of arbutin is often limited by its lower potency and potential for causing irritation or toxicity at higher concentrations [2].

The potential of natural products as anti-melanogenic agents is increasingly being recognized due to their diverse biological activities and favorable safety profiles. Several studies have demonstrated the efficacy of natural compounds in inhibiting melanin synthesis. Previous studies have shown that Chi-Bai-San could effectively inhibit melanogenesis by targeting CREB, ZEB2, β-linker protein and MITF/tyrosinase signaling pathway without causing significant toxicity [3]. The extracts from *Nymphaea hybrid* have demonstrated comparable effectiveness to established treatments like tretinoin without cytotoxic effects [4]. Additionally, plant-derived components, including those from *Safflower* and *Arnica montana*, have shown inhibitory effects on melanogenesis, suggesting their potential therapeutic applications [5] and indicating their potential value in anti-melanogenesis research. The molecular mechanisms underlying the anti-melanogenic effects of natural products often involve the suppression of MITF and its downstream effectors, TYR, TRP-1, and TRP-2, through pathways such as α-MSH-MC1R, Wnt, NO, PI3K/Akt, and MAPK [6]. The PI3K/Akt signaling pathway, upon activation by extracellular signals or intracellular cyclic adenosine monophosphate (cAMP), leads to Akt-mediated glycogen synthase kinase 3β (GSK3β) deactivation, reducing tyrosinase expression. Similarly, the MAPK signaling pathway is activated by stem cell growth factor (SCF) or vasoconstrictor peptide (EDN-1), which causes Ras to activate the mitogen-activated protein kinase (MAPK) cascade [7], which leads to proteasomal degradation of the phosphorylated MITF, which down-regulates melanogenesis [8].

*Reed Rhizome* (*Phragmitis Rhizoma*), the dried rhizome of *Phragmites communis* Trin, is employed in traditional Chinese medicine for a variety of ailments, including pneumonia, diabetes, and acne [9]. Modern pharmacological studies demonstrated that *Reed Rhizome* extract has various activities such as antioxidant, α-glucosidase inhibition [10] and anti-inflammatory [11]. Its safety has been validated through rigorous genotoxicity assessments [12]. A diverse array of bioactive compounds identified within *Reed Rhizome*, particularly terpenoids, flavonoids, and phenols, are known for their anti-melanogenic properties [5]. Given the reported efficacy of young reed leaf extracts in modulating the CREB/MITF/tyrosinase pathway and their antioxidant capabilities [13], we posited that *Reed Rhizome* root extract might also possess anti-melanogenic activity.

The zebrafish (*Danio rerio*) is a freshwater vertebrate that is easily bred in a laboratory environment and has rapid embryonic development. Its transparency during early embryonic stages enables straightforward observation of organ development and melanin deposition under a microscope [14]. Therefore, it is an ideal model for screening and researching anti-melanogenesis agents. This study employed the zebrafish model, an established *in vivo* system for the rapid assessment of melanin synthesis and pigment pattern formation, to evaluate the anti-melanogenesis effects of these natural compounds.

Herein, we report the discovery of a novel natural compound belonging to phenylpropanoid glycosides, (1'S, 2'S)-syringyl glycerol 3'-O-β-D-glucopyranoside, also named compound **5**, which exhibited a significant inhibitory effect on melanogenesis, surpassing the efficacy of arbutin. This finding prompted a comprehensive investigation into the anti-melanogenic mechanisms of compound **5**, including *in vivo* and *in vitro* assays, network pharmacological analysis, and gene expression studies. Our results not only highlight the potential of compound **5** as a superior melanogenesis inhibitor but also provide insights into its molecular targets and the underlying pathways involved in its anti-melanogenic effects. The identification of key regulatory genes affected by compound **5** offers a new perspective on the modulation of melanin synthesis and presents a promising therapeutic avenue for the treatment of hyperpigmentation disorders.

## 2 MATERIALS AND METHODS

### 2.1 Materials and Instruments

Wild strain Tu zebrafish was provided by the laboratory of Prof. Jingwei Xiong, Peking University. *Reed Rhizome* root extract components were provided by Prof. Yanfang Su's team at the School of Pharmaceutical Science and Technology Tianjin University. *Reed Rhizome* was collected from Baiyangdian, Baoding City, Hebei Province, China, in April 2018 and identified as the dried root of *Phragmites communis* Trin. by Prof. Tianxiang Li at Tianjin University of Traditional Chinese Medicine. The sample (201804001) was stored at the Laboratory of Natural Medicinal Chemistry, College of Pharmaceutical Science and Technology, Tianjin University. Fresh potatoes (*Solanum tuberosum* L.), 98% arbutin and 98% levodopa (Heowns Biochem Technologies Co., Ltd., Beijing, China), total RNA extract (Gene-Protein Link Biotechnology Co., Ltd., Beijing, China), Hifair^®^ III 1st Strand cDNA Synthesis SuperMix (gDNA digester plus) and Hieff^®^ RT-qPCR SYBR Green Master Mix (No Rox) (Yeasen Biotechnology Shanghai Co., Ltd., Shanghai, China), the primers and the endogenous gene *gapdh* (Suzhou Jinweizhi Biotechnology Co., Ltd., Suzhou, Jiangsu Province, China) were used.

The SMZ800N *in vivo* fluorescence microscope (Nikon Corporation, Tokyo, Japan), the HIM multifunctional enzyme labeler (Bio Tek Instruments, Inc., Vermont, USA), the DS-11 Ultra-Micro Spectrophotometer (DeNovix Inc., Wilmington, Delaware, USA) and the fluorescence quantitative PCR instrument (Bio-Rad Laboratories, Hercules, California, USA) were used in this experiment.

All other materials and instruments were high- grade and commercially available.

### 2.2 Extraction and Isolation of Compound **Monomers**

The dried *Reed Rhizome* was extracted by hot reflux extraction, and the resulting extract was dispersed in an appropriate amount of distilled water and extracted sequentially. The aqueous layer obtained from the extraction was adsorbed by D101 large pore adsorbent resin and then eluted. The ethanol-eluting portion was dissolved in methanol, and the soluble material was upsampled. A silica gel column was eluted with the eluent, and the combined samples were analysed by TLC to obtain seven streams of Frs1-Frs7. Frs5 was then placed on an ODS column and gradient eluted to obtain Frs1-Frs23. Finally, Frs16 was isotropically eluted by reversed-phase HPLC to obtain amorphous solids. The compound monomers were isolated and purified by elution on a preparative column with the eluents [15].

### 2.3 Zebrafish Breeding and Embryo Collection

The zebrafish used was the wild-type Tu strain, which was bred following the conditions outlined in Technical Conditions for Feeding Zebrafish for Experimental Use (DB32/T 3979-2021). After mating zebrafish according to the routine procedure, we selected viable embryos under a microscope and flushed them with E3 culture solution (5 mM NaCl, 0.17 mM KCl, 0.4 mM CaCl_2_, and 0.33 mM MgSO_4_). Then, these embryos were placed in an incubator at 28.5°C for further experimentation. All animal procedures were approved by the Animal Ethics Committee of Tianjin University (protocol code TJUE-2023010).

### 2.4 Zebrafish Embryo Exposure Experiments

The zebrafish embryo exposure experiments consisted of two stages: the pre-experiment stage and the formal experiment stage. Zebrafish embryos at 12 hours post-fertilization (hpf) were selected for the experiments and placed in 24-well plates.

For the pre-experiment, two parallel groups were established. Each well of the groups was filled with 1 mL of either pre-prepared *Reed Rhizome* extract solution or arbutin solution at varying concentrations ranging from 0 μg/mL to 2000 μg/mL. For the formal experiments, the groups were divided into three: the test drug group, the positive control group (arbutin), and the blank control group (E3), each with two parallel experimental wells. Each group received 1 mL of either extract, arbutin, or E3 culture solution, with the solution concentration chosen based on the optimal effective concentration of extract tested in the pre-experiment. For further breeding, 24-well plates were placed in a constant-temperature incubator. The above solutions were prepared using E3 culture solution.

At 48 hpf, the body surface pigmentation of zebrafish embryos was observed and photographed under an *in vivo* microscope.

### 2.5 Determination of Melanin Inhibition in Zebrafish

The *in vivo* inhibition of melanin was determined by cleavage with NaOH [16].

Three hundred 12 hpf zebrafish embryos were divided randomly into 12 wells of a 24-well plate. The 12 wells were divided into 3 groups (E3 culture solution, 1500 μg/mL arbutin and 1500 μg/mL compound **5**), and each well in each group received 1 mL solution. Incubate at 28°C for 48 h in the absence of light. Next, 20 zebrafishes were selected per well, flushed with PBS and subsequently transferred to EP tubes containing 150 μL of a sodium deoxycholate solution (5 g/L). The homogenate was prepared by using an ultrasonic crusher at a low temperature and then cryo-centrifuged [16]. The supernatant was discarded, and the precipitate was mixed with 150 μL of 1 mol/L NaOH solution. The solvent control group consisted of untreated zebrafish, while the blank control group was composed of the E3 solution. Each sample was measured for absorbance at a wavelength of 490 nm using 100 μL [17].

The rate of melanin synthesis inhibition was calculated using the following Eq. (**1**)

Melanin synthesis inhibition rate 


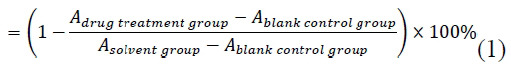


### 2.6 Determination of *in vivo* Tyrosinase Inhibition in Zebrafish

The *in vivo* tyrosinase inhibitory activity was determined using the dopa oxidation method [16].

Three hundred 12 hpf zebrafish embryos were divided randomly into 12 wells of a 24-well plate. The 12 wells were divided into 3 groups (E3 culture solution, 1500 μg/mL arbutin and 1500 μg /mL compound **5**), and each well in each group received 1 mL solution. Incubate at 28°C for 48 h in the absence of light. Next, 20 zebrafishes were selected per well, flushed with PBS and subsequently transferred to EP tubes containing 150 μL of a sodium deoxycholate solution (5 g/L). The homogenate was then prepared using an ultrasonic crusher at low temperature and cryo-centrifuged [17]. 100 μL of the supernatant was added to an EP tube, followed by the addition of 100 μL of levodopa solution (5 μmol/L) and incubation at 37°C for 1 h. The solvent control group consisted of untreated zebrafish, while the blank control group was composed of the E3 solution. Each sample was measured for absorbance at a wavelength of 475 nm using 100 μL.

The rate of *in vivo* tyrosinase inhibition was calculated using the same formula as (1)

### 2.7 Determination of *in vitro* Tyrosinase Inhibition in Potato

Potatoes are rich in tyrosinase and can be used as a source of tyrosinase *in vitro*. To exclude other complicating factors *in vivo*, the tyrosinase inhibitory activity was determined *in vitro* using the mushroom tyrosinase dopa oxidation method [18].

To prepare the crude potato tyrosine enzyme solution, 4 g of the frozen potato blocks were ground with phosphate buffer (20 mL, pH 7.2-7.4) Then, the mixture was taken up with a dropper and dispensed into EP tubes. They were immediately centrifuged and the supernatant was the crude enzyme solution, which was then stored at 4°C.

PBS buffer, L-dopa solution (1.48 mg/mL), crude potato tyrosine enzyme solution, drugs (compound **5** or arbutin) and purified water were mixed according to (Table **1**) to formulate different reaction systems. The reaction was carried out at room temperature without exposure to light for 40 min. The absorbance was measured at 475 nm. Three parallel experimental groups were set up for each sample, and each group was measured three times. The average value was used to calculate the tyrosinase inhibition rate [19].

The *in vitro* tyrosinase inhibition rate was calculated using the following Eq. (**2**)

The *in vitro* tyrosinase inhibition rate


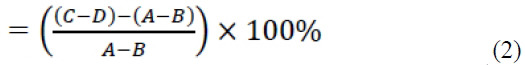


The following Table **1** shows the reaction systems involving A, B, C, and D in the above equation.

### 2.8 Network Pharmacology Analysis

Swiss Target Prediction (http://www.swisstarget-prediction.ch/) and Super Pred (https://prediction.charite.de/) were used to predict compound **5** potential target genes. Duplicate genes were removed to obtain the final list. (1) The GeneCards database (https://www.genecards.org) was screened for melanogenesis-related targets using the term “melanin”. (2) Gene and protein names were standardized using the UniProt database (http://www.uniprot.org/). (3) The Venny tool (http://bioinformatics.psb.ugent.be/webtools/Venn/) was used to cross-reference the compound **5** targets and the melanin targets to obtain their cross-targets, *i.e.* potential targets of compound **5** for melanogenesis.

Based on the cross-targets, the protein-protein interaction (PPI) network was constructed in the STRING database (http://string-db.org/) with the species restriction set to “*Homo sapiens*” and the minimum interaction threshold set to “medium confidence” (>0.4). Importing the PPI network data into Cytoscape 3.7.1, the individual nodes were hidden, and the topology analysis was performed using the software's “Network analyzer”. The core targets were then identified based on a degree value that exceeded the average.

Metascape (http://metascape.org/gp/index.html) was used to perform Gene Ontology (GO) and Kyoto Encyclopedia of Genes and Genomes (KEGG) enrichment analysis of the core targets.

Molecular docking was performed between ligand compound **5** and the top three potential core target proteins. The chemical structure of the ligand was visualized using InDrawforWeb (https://indrawforweb.integle.com/). The PDB Formats of the target proteins were obtained from the Protein Data Bank database (PDB, https://www.rcsb.org) with the restriction of *Homo sapiens*. BIOVA Discovery Studio 2019 Client was used to carry out the molecular docking process and its visualization. The process involved the following steps: 1) Converting the ligand from a two-dimensional structure to a three-dimensional structure. 2) Removing the original ligand and redundant water molecules from the target protein, adding the missing hydrogens, and predicting binding sites of the protein pockets. 3) Calculate the LibDock score and the binding energies to indicate the binding degree of the ligand to the receptor. 4) Visualizing the docking results.

### 2.9 Determination of Relative Gene Expression in Zebrafish

Total RNA was extracted from zebrafish embryos using TRIzol reagent. The concentration was measured using a spectrophotometer. The initial cDNA strand was produced using the Hifair^®^ III 1st Strand cDNA Synthesis SuperMix for RT-qPCR (gDNA digester plus) Reverse Transcription Kit. The reverse transcription program was set up as follows: 25°C for 5 minutes, 55°C for 15 minutes, and 85°C for 5 minutes. The cDNA was amplified using the designed primers and Hieff^®^ RT-qPCR SYBR Green Master Mix (No Rox) kit. qPCR program sets as follows: (1) Heat the sample to 95°C for 5 minutes; (2) Denature the DNA by heating to 95°C for 10 seconds; (3) Cool it down to 60°C for 20 seconds; (4) Extend the strand by heating to 72°C for 20 seconds. Repeat steps (2) through (4) for a total of 40 cycles and the final extension step at 65°C for 5 seconds. The primer sequences for each gene in this study were listed in Table **S1**.

### 2.10 Statistical Analysis

The results were analyzed using GraphPad Prism (version 8.0 for Windows, GraphPad Software, Boston, Massachusetts, USA) software and expressed as the mean ± standard deviation. Differences between means were assessed using one-way ANOVA, and *p* < 0.05 was considered statistically significant.(supplementary material).

## 3 RESULTS

### 3.1 Compound 5 Significantly Inhibited Melanogenesis in a Zebrafish Model

In our initial screening of *Reed Rhizome* extracts for anti-melanogenesis activity, we treated zebrafish embryos with seven different extracts and observed melanin deposition under the microscope. The chemical composition of the aqueous fraction of the ethanolic extract of *Reed Rhizome* extracted by organic solvents was investigated in the present study. A total of eight compounds were isolated and identified, as follows: cuneataside D (compound **1**), (1'R, 2'R)-syringyl glycerol 3'-O-β-D-glucopyranoside (compound **2**), (1'R, 2'R)-guaiacyl glycerol 3'-O-β-D-glucopyranoside (compound **3**), (1'S, 2'R)-guaiacyl glycerol 3'-O-β-D-glucopyranoside (compound **4**), (1'S, 2'S)-syringyl glycerol 3'-O-β-D-glucopyranoside (compound **5**), (1'S, 2'S)-guaiacyl glycerol 3'-O-β-D-glucopyranoside (compound **6**), (1'R, 2'S)-syringyl glycerol 3'-O-β-D-glucopyranoside (compound **7a**) and (1'S, 2'R)-syringyl glycerol 3'-O-β-D-glucopyranoside (compound **7b**). The aforementioned compounds were designated as compounds 1-7b, with compound **1** representing the phenolic glycosides and the remaining compounds representing the phenylpropanoid glycosides. The chemical structures of these extracts are shown in Fig. (**1A**), with arbutin as a positive control.

**Fig. (1):**
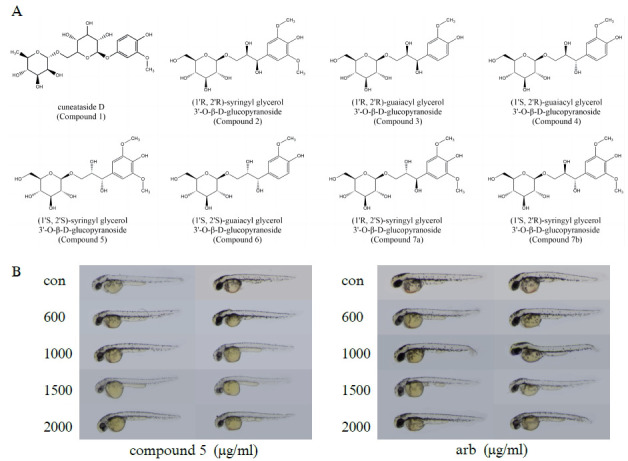
Results of preliminary screening experiment. (**A**) Chemical structures of the seven natural products candidates. Compound **1** belongs to phenolic glycosides and compounds **2**-**7** belong to phenylpropanoid glycosides. (**B**) The anti-melanogenesis effect of compound **5** at 1500 μg/mL was better than that of arbutin at the same concentration.

Zebrafish embryos were exposed to different concentrations of *Reed Rhizome* extracts and the results are shown in Fig. (**S1**). Among the tested extracts, 1000 μg/mL compound **2**, 800 μg/mL compound **6** and 600 μg/mL compound **7** showed significant teratogenic effects on zebrafish. Although compounds **1**, **3** and **4** did not exhibit any teratogenic effects on zebrafish, they also had no significant anti-melanogenesis effect at the experimental concentrations. It is important to note that with 1500 μg/mL, compound **5** significantly inhibited melanin deposition in zebrafish embryos. Its anti-melanogenesis effect was superior to that of arbutin at the same concentration. Furthermore, compound **5** did not have any teratogenic or lethal effects on zebrafish embryos (Fig. **1B**). Consequently, compound **5** was selected for further investigation as a potential anti-melanogenic agent.

We used 1H NMR spectrum to compare the structural features of compound **5** with known literature data (Table **S2**) and plotted 1H NMR spectrum (Fig. **S2**) [20].

### 3.2 Compound 5 Significantly Inhibited Melanogenesis and Tyrosinase Both *in vivo* and *in vitro*

**Fig. (2):**
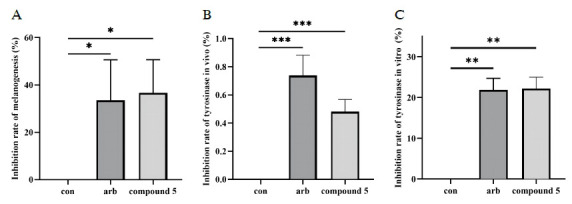
(**A**-**C**) Compound 5 significantly inhibited melanogenesis and tyrosinase activity both *in vivo* and in potato *in vitro* ***p < 0.001, **p < 0.01 and *p < 0.05 as compared with the control group.

Tyrosinase is a key enzyme in the melanogenic process. To measure melanin and tyrosinase inhibition separately, absorbance was measured at 490 nm using NaOH cleavage and at 475 nm using dopa oxidation, respectively. Finally, we calculated the inhibition rate of melanogenesis and tyrosinase activity both *in vivo* and *in vitro* (Fig. **2**).

*In vivo* assays using zebrafish larvae showed that compound **5** at a concentration of 1500 μg/mL significantly reduced melanogenesis compared to the untreated control group. The observed inhibition rate was 36.66% ± 14.00% (*p* < 0.05), which was comparable to the rate achieved by arbutin, a known melanogenesis inhibitor, which had an inhibition rate of 33.52% ± 17.08% at the same concentration (Fig. **2A**).

*In vivo* analysis demonstrated that both arbutin and compound **5** significantly decreased tyrosinase activity in zebrafish embryos. Arbutin showed a higher inhibition rate of 73.92% ± 11.64% compared to compound **5**, which showed an inhibition rate of 48.26% ± 6.94%, both of which were statistically significant (*p* < 0.001) when compared to the control (Fig. **2B**).

*In vitro* experiments using a crude potato tyrosinase solution treated with 1500 μg/mL of either arbutin or compound **5** also showed significant inhibition of tyrosinase activity. The inhibition rates were 21.85% ± 4.90% for arbutin and 22.16% ± 4.82% for compound **5** (Fig. **2C**).

The above results highlight the potential of compound **5** as a potent inhibitor of melanin and tyrosinase synthesis.

### 3.3 Network Pharmacological Analysis Identified HSP90AA1, HRAS and PIK3R1 as Potential Targets of Compound **5**

Compound 5 has demonstrated a more potent inhibitory effect on melanin synthesis than the established positive control, arbutin. Despite its superior efficacy in reducing melanin content, compound **5** exhibits a less inhibitory effect on tyrosinase activity compared to arbutin. This discrepancy suggests that compound **5** may exert its anti-melanogenic effects through a complex, multifaceted mechanism that extends beyond the inhibition of tyrosinase alone. To clarify the underlying pathways and molecular interactions, the study conducted a thorough network pharmacological analysis of compound **5**. The results are illustrated in Fig. (**3A**-**G**).

**Fig. (3):**
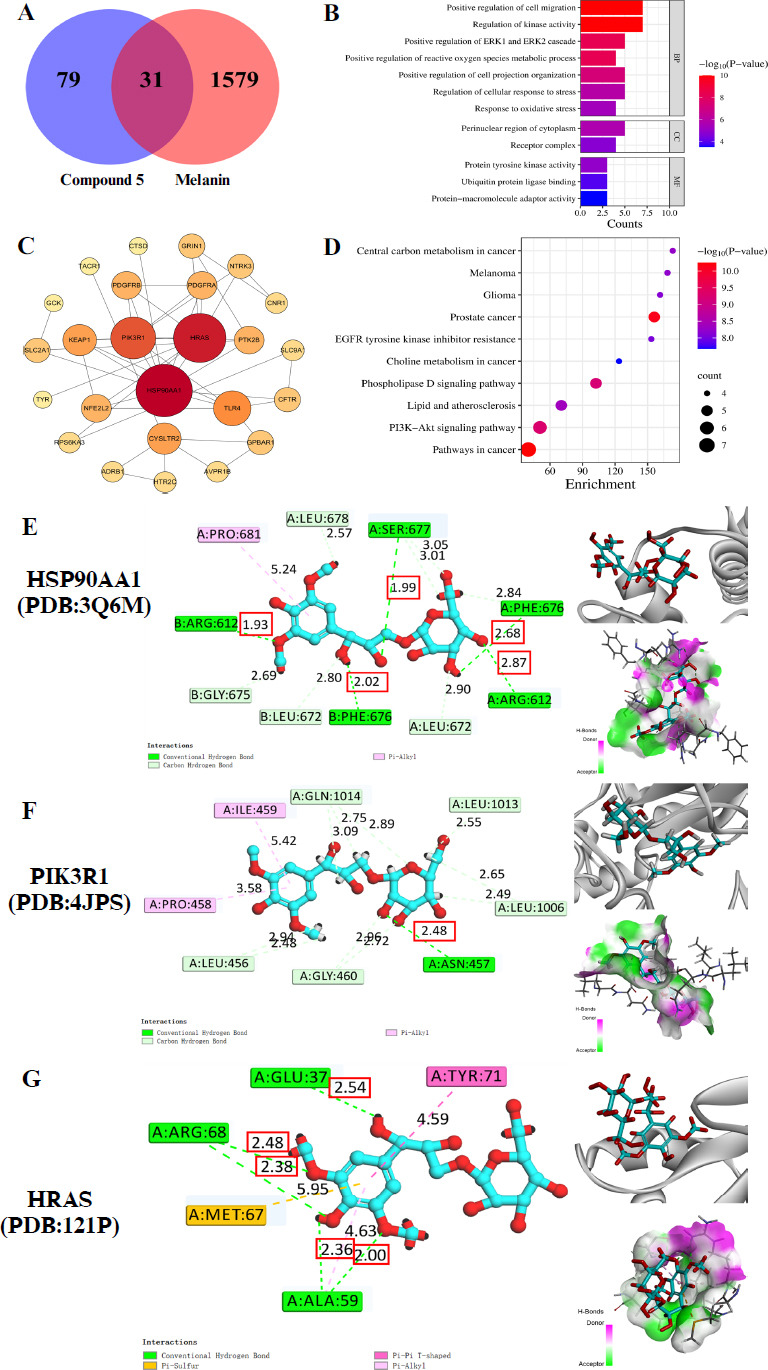
Compound 5 may act on HSP90AA1 or (and) HRAS or (and) PIK3R1 to inhibit melanogenesis. (**A**) The Venn diagram of compound **5** and melanin targets. (**B**) PPI of cross-targeted proteins, with the outermost circle of degree 1-3, the middle circle of 4-7, and the smallest circle of 8-11. (**C**) GO analysis of genes encoding proteins targeted by compound **5**. (**D**) KEGG analysis of genes encoding proteins targeted by compound **5**. (**E-G**) 2D and 3D docking diagrams between compound **5** and core target proteins. The boxes represented amino acid residues, the letter “A” or “B” preceding the amino acid abbreviation represented the A or B chain of the protein, and the number following it represented the serial number of the amino acid in the polypeptide chain.

A total of 110 potential targets of compound **5** were obtained from SwissTargetPrediction and Super Pred. One thousand six hundred ten melanin-related targets were obtained from GeneCards. Cross-referencing these datasets revealed 31 common targets, suggesting their potential role in the anti-melanogenic effects of compound **5** (Fig. **3A**).

The PPI network was established based on the cross-target genes and was analysed by STRING and Cytoscape 3.7.1 for topological analysis. The PPI network showed 25 shared proteins and 46 edges between compound **5** and melanogenesis. The average degree was 3.68, with 10 targets having a degree higher than the average. These genes included HSP90AA1 (heat shock protein 90 alpha family class A member 1), HRAS (HRas proto-oncogene, GTPase), PIK3R1 (phosphoinositide 3-kinase regulatory subunit 1), TLR4 (toll-like receptor 4), KEAP1 (kelch like ECH associated protein 1), CYSLTR2 (cysteinyl leukotriene receptor 2), NFE2L2 (nuclear factor, erythroid 2 like 2), PDGFRB (platelet-derived growth factor receptor beta), PDGFRA (platelet-derived growth factor receptor alpha), PTK2B (protein tyrosine kinase 2 beta) (Fig. **3B**) (Table **S3**), suggesting they were the core targets of compound **5** intervention in melanogenesis.

To predict the mechanism of compound **5** on melanogenesis inhibition, Metascape was used to perform GO and KEGG enrichment analysis on the 10 core targets (Fig. **3C-D**). The GO enrichment analyses (Fig. **3C**) yielded a total of 156 entries in terms of biological process (BP), cellular component (CC), and molecular function (MF). In BP-enriched pathway, there were 143 entries and 6 clusters. The target proteins were mainly involved in the positive regulation of cell migration, regulation of kinase activity, and positive regulation of the ERK1 and ERK2 cascade. In CC-enriched pathway, there were 2 entries and 2 clusters. The target proteins were mainly divided into the perinuclear region of the cytoplasm and the receptor complex. In the MF-enriched pathway, there were 11 entries and 3 clusters. The target proteins were mainly involved in protein tyrosine kinase activity, ubiquitin protein ligase binding and protein-macromolecule adaptor activity. The KEGG enrichment analysis yielded 34 entries, of which the top 10 entries were screened (Fig. **3D**). These included cancer-related signaling pathways (including melanoma), the PI3K-Akt signaling pathway, atherosclerosis, *etc*.

### 3.4 Compound 5 Exhibited a Robust Docking Activity with the Core Target Proteins

To validate the interaction between compound **5** and the core targets, we performed molecular docking of compound **5** with the first three core targets, HSP90AA1 (PDB:3Q6M), HRAS (PDB:121P), and PIK3R1 (PDB:4JPS). The results indicated that compound **5** was able to bind to the docking pockets of HSP90AA1, HRAS and PIK3R1 (Fig. **3E**) with LibDock scores of 125.179, 130.63, and 133.077, respectively. According to the two-dimensional interaction diagram (Fig. **3E**), compound **5** bound to the binding pocket of HSP90AA1 by forming five hydrogen bonds with SER-677, A-PHE-676, A-ARG-612, B-PHE-676 and B-ARG-612 (at the distances of 1.99, 2.68, 2.87, 2.02, 1.93 Å respectively). Similarly, compound **5** bound to the binding pocket of HRAS by forming 5 hydrogen bonds with GLU-37 (at a distance of 2.54 Å), ALA-59 (at the distances of 2.36 Å and 2.00 Å, respectively) and ARG-68 (at the distances of 2.48 Å and 2.38 Å, respectively). Additionally, compound **5** bound to the binding pocket of PIK3R1 by forming one hydrogen bond with ASN-457 at a distance of 2.48 Å. These interactions highlight the potential of compound **5** to regulate the activity of these proteins, providing a mechanistic understanding of its anti-melanogenic effects. Meanwhile, considering the impact of spatial conformation, we also docked and calculated binding energies for the isomers of compound **5**, *i.e.*, compound **2**, compound **7a** and compound **7b** (Table **S4**).

### 3.5 Compound 5 Upregulated *hsp90aa1.1*, *Hrasa*, and *pik3r1* to Inhibit Melanogenesis

To test the network pharmacological analyses and elucidate the molecular mechanisms underlying the anti-melanogenic effects of compound **5**, we evaluated the expression levels of key melanogenic genes *hsp90aa1.1*, *hrasa*, and *pik3r1* in zebrafish after exposure to compound **5** and compared them to the effects of arbutin.

**Fig. (4):**
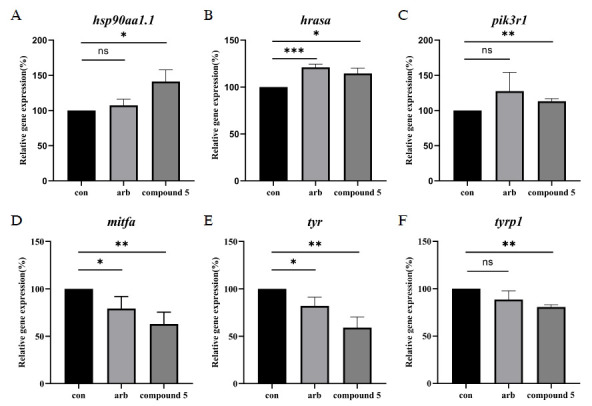
(**A-F**) Compound 5 up-regulated the gene expression of *hsp90aa1.1*, *hrasa* and *pik3r1,* while down-regulating the gene expression of *mitfa*, *tyr* and *tyrp1* in zebrafish larvae. ***p < 0.001, **p < 0.01 and *p < 0.05 as compared with the control group.

HSP90AA1, a molecular chaperone, plays a pivotal role in various cellular processes, both physiological and pathological. It facilitates the folding and activation of client proteins [21]. Treatment with compound **5** resulted in a significant upregulation of *hsp90aa1.1*, with expression levels reaching 141.17% ± 16.67% relative to control, indicating its potential role in stabilizing molecular substrates (Fig. **4A**). It is noteworthy that HSP90AA1 plays a role in the stability of Akt within the PI3K signaling pathway. This suggested that compound **5** exerted a positive regulatory effect on the PI3K signaling pathway [21, 22]. Another gene of significance in the process of melanogenesis is HRAS, which belongs to the RAS protein family, which also includes KRAS and NRAS. These proteins are GTPases that activate MAPK-dependent signaling pathways, which are essential for melanin production [23]. The results demonstrate that compound **5** upregulated *hrasa* expression to 114.48% ± 5.74% of control levels, suggesting a regulatory effect on the MAPK signaling pathway and, consequently, melanogenesis (Fig. **4B**). PIK3R1, the predominant regulatory subunit of PI3K [24], is a key mediator in the PI3K/AKT signaling cascade, which is implicated in α-MSH-induced melanogenesis [25]. The results showed that compound **5** up-regulated *pik3r1* to 113.12% ± 3.73% relative to the control, further supporting the positive regulatory influence of compound **5** on this pathway (Fig. **4C**).

Additionally, *mitfa*, *tyr*, and *tyrp1*, which are directly involved in the melanin synthesis pathway, were also measured. MITF is a transcription factor that directly activates genes that regulate melanocyte differentiation, proliferation, and survival. Its downstream genes, such as *tyr* and *tyrp1*, also affect melanin synthesis [26]. After treatment with compound **5** to zebrafish embryos, the relative gene expression levels of *mitfa*, *tyr*, and *tyrp1* were 62.93% ± 12.44%, 59.08% ± 11.29% and 80.87% ± 2.33%, respectively, suggesting that compound **5** may affect the expression of downstream melanin synthesis-related genes through its action on upstream genes.

In summary, the study demonstrated that compound **5** was capable of inhibiting melanogenesis by up-regulating the expression of *hsp90aa1.1*, *hrasa* and *pik3r1*, and subsequently down-regulating the expression of *mitfa*, *tyr* and *tyrp1*. In comparison to arbutin, the zebrafish treated with compound **5** exhibited a more pronounced decrease (*p* < 0.01) in *mitfa* and its downstream melanogenesis-related genes, thereby indicating a superior anti-melanogenesis effect of compound **5**. Furthermore, arbutin treatment demonstrated no significant effect on the expression of *hsp90aa1.1* and *pik3r1* in comparison to the control group, indicating that the regulatory mechanisms of compound **5** and arbutin are distinct. These results highlight the potential of compound **5** as an agent with anti-melanogenic properties. (Fig. **4D**-**F**)

## 4 DISCUSSION

The search for efficacious and safe anti-melanogenic agents is of significant interest in the field of dermatology due to the prevalence of pigmentary disorders. This study presents *Reed Rhizome* extract compound **5** as a novel inhibitor of melanogenesis, demonstrating superior efficacy to the well-used agent arbutin in both *in vivo* and *in vitro* models.

Our initial screening revealed that compound **5** significantly reduces melanin deposition in zebrafish embryos without inducing teratogenic effects, a notable advantage over other extracts tested. The *in vivo* and *in vitro* assays confirmed the potent inhibitory effect of compound **5** on melanogenesis and tyrosinase activity, which is a pivotal enzyme in the melanin synthesis pathway. However, while arbutin showed a higher tyrosinase inhibition rate *in vitro*, compound **5** was more effective in reducing melanin content *in vivo*, suggesting different mechanisms of action for these two compounds.

The previous studies indicated that arbutin is a tyrosinase inhibitor [27] and also reduces α-MSH-induced expression of *TYR* and *MITF* within B16F10 [28, 29]. In agreement with our experiments, arbutin significantly reduced tyrosinase activity and decreased the expression levels of *mitfa* and *tyr* genes in zebrafish. Unlike arbutin, the results of the network pharmacology analysis showed that compound **5** has a multitarget effect in inhibiting melanin production. Among them, HSP90AA1, HRAS, and PIK3R1 were the core target proteins, and PI3K-Akt is one of the most important signaling pathways. Fig. (**4**) showed that compound **5** up-regulated the expression of zebrafish-related genes *hsp90aa1.1*, *hrasa*, and *pik3r1*, which was consistent with the expected results of network pharmacology.

The protein encoded by *pik3r1* (p85α) is a regulatory subunit of PI3K that mediates the activation of the catalytic subunit of PI3K, P110, through direct interaction with phosphotyrosine residues of activated growth factor receptors or adaptor proteins. The presence of PIK3R1 has been demonstrated to increase PI3K activity [30, 31], thereby activating the PI3K-Akt pathway. Research has shown that the PI3K-Akt axis is a major signaling pathway in the regulation of melanogenesis. Activation of PI3K results in the phosphorylation of Akt at Ser473, which in turn leads to the inactivation of GSK3β through its phosphorylation at Ser9. This prevents GSK3β from cooperating with MITF to activate the tyrosinase promoter and regulate melanogenesis [7, 32]. Consistent with our results, decursin and 3, 4-dihydroxybenzoylacetone (DBL) can activate the PI3K-Akt cascade, promoting the phosphorylation of Akt and GSK3β, which inhibits melanogenesis [33, 34].

HSP90AA1 functions as a molecular chaperone, stabilizing signaling molecules such as PI3k and Akt [35]. Therefore, activation of HSP90AA1 will promote Hsp90AA1-Akt protein-protein interaction [36], which significantly enhances the activity of Akt and reduces melanogenesis *via* PI3K-Akt.

Additionally, the *hrasa*-encoded protein HRas is a member of the Ras family. Accumulation of HRas could activate the Ras-Raf-MEK-ERK pathway *via* the HRas-GTP complex [37, 38], which is also involved in the regulation of melanogenesis. The Ras-Raf-MEK-ERK cascade activates ERK [39], which in turn phosphorylates MITF, leading to its degradation by the proteasome [7, 32]. It has been reported that the transfection of H-ras into normal mouse melanocytes has been found to inhibit melanogenesis [40].

Taken together, compound **5** upregulates the expression of *hsp90aa1.1*, *hrasa*, and *pik3r1* while concurrently downregulating *mitfa*, *tyr*, and *tyrp1*, which are directly involved in melanin synthesis. MITF is the main transcription factor in melanogenesis and a target of multiple melanogenic signaling pathways, including the PI3K-Akt and Ras-Raf-MEK-ERK pathways mentioned previously. It acts as a transcriptional activator to regulate the expression of key downstream melanogenesis-related genes, such as *tyr* and *tyrp1* [41]. The genes responsible for melanin synthesis, *mitfa*, *tyr*, and *tyrp1* were significantly downregulated by compound **5**. This effect was better than that of the positive control, arbutin. Previous studies have shown that up-regulating the expression of core genes in the PI3K-Akt and Ras-Raf-MEK-ERK signaling pathways can down-regulate the expression of *mitf* and its downstream genes [42]. This is consistent with our findings. Therefore, our analysis suggests that compound **5** inhibits melanin synthesis by regulating PI3K-Akt and Ras-Raf-MEK-ERK signaling pathways. This dual regulatory effect on gene expression is particularly significant as it provides a possible explanation for the superior anti-melanogenic activity of compound **5** compared to arbutin, which did not affect the expression levels of *hsp90aa1.1* and *pik3r1*. Among the samples tested in this study, compound **5** exhibited the most significant melanin inhibitory activity in the zebrafish model. Compound **5** belongs to the phenylpropanoids, whose parent nucleus consists of a benzene ring linked to three straight-chain carbons. The observed melanin-inhibitory effect may be attributed to the substituent groups on the benzene ring and the absolute configuration of the three-carbon chain.

However, while the current study elucidates the potential of compound **5** as an anti-melanogenic agent, further investigation is required to elucidate its specificity and potential off-target effects. With regard to specificity, the targeting of Compound **5** on PI3K, Akt, HRAS and related signalling molecules could be validated in future studies by using specific inhibitors and gene knockdown approaches. With regard to off-target effects, we have initially demonstrated that compound **5** has no significant effect on the morphogenesis development and normal survival of zebrafish embryos at the concentrations employed in our experiments. Further comprehensive *in vivo* studies and off-target screening are warranted to fully elucidate the safety profile of compound **5**. Additionally, the exploration of its effects on other melanin-related pathways and potential anti-inflammatory or antioxidant properties could provide a comprehensive view of its therapeutic potential.

## CONCLUSION

In conclusion, our findings introduced compound **5** as a potent and multifaceted inhibitor of melanogenesis with a unique mechanism of action. The promising results from this study pave the way for future pharmacological developments and clinical trials, with the potential for compound **5** to emerge as a leading treatment for hyperpigmentation disorders.

## Figures and Tables

**Table 1:** Reaction system for *in vitro* tyrosinase activity inhibition assay.

**Materialistic(mL)**	**A**	**B**	**C**	**D**
PBS	0.5	0.5	0.5	0.5
L-dopa	0.5	0.5	0.5	0.5
Tyrosinase solution	0.5	0	0.5	0
Drug	0	0	0.5	0.5
Purified water	0.5	1	0	0.5
Total reaction volume	2	2	2	2

**Notes:** A: represents negative control without drug but with tyrosinase. B: represents blank control without drug and tyrosinase. C: represents blank control containing drug and tyrosinase. D: represents blank control containing drug but not tyrosinase.

## Data Availability

All data generated or analysed during this study are included in this published article.

## LIST OF ABBREVIATIONS

GSK3β: Glycogen Synthase Kinase 3β
MAPK: Mitogen-activated Protein Kinase
