# Supplementary material for: Phenolic Glycoside Monomer from Reed Rhizome Inhibits Melanin Production via PI3K-Akt and Ras-Raf-MEK-ERK Pathways
Source: Curr Med Chem. 2024 Sep 27;32(38):8664–77. doi: 10.2174/0109298673341645240919072455 (PMC12645109; doi:10.2174/0109298673341645240919072455)
Supplement: Supplementary file 1 [file CMC-32-38-8664_SD1.pdf]

Supplementary Material

Phenolic Glycoside Monomer fromReed Rhizome Inhibits Melanin Production *via* PI3K-Akt and Ras-Raf-MEK-ERK Pathways

Meijun Pang[1,2,3,#], Hong Yao[1,2,3,#], Kechen Bao[1,2,3,#], Ruitian Xu[1,2,3], Rongjiao Xi[1,2,3], Rui Peng[1,3,4], Hui Zhi[1,3,4], Kuo Zhang[1,3,4], Runnan He[1,3,4], Yunfei Du[4], Yanfang Su[4], Xiuyun Liu[1,3,4,*] and Dong Ming[1,3,4,*]

[1] Medical School, Tianjin University, 92 Weijin Road, Nankai District, Tianjin, 300072, China; [2]Haihe Laboratory of Brain -Computer Interaction and Human-Machine Integration, Tianjin, 300072, China; [3]State Key Laboratory of Advanced Medical Materials and Devices, Tianjin, 300072, China; [4]Department of Neurosurgery, Tianjin Medical University General Hospital, 154 Anshan Street, Heping District, Tianjin, 300052, China

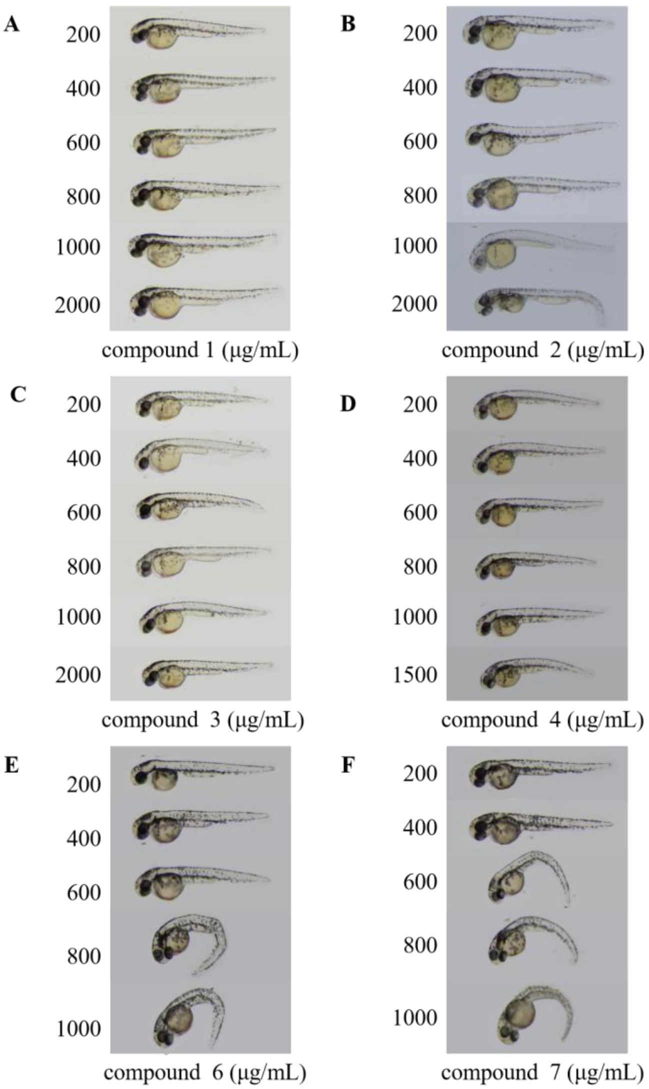

Fig. (S1). Results of preliminary screening experiment.

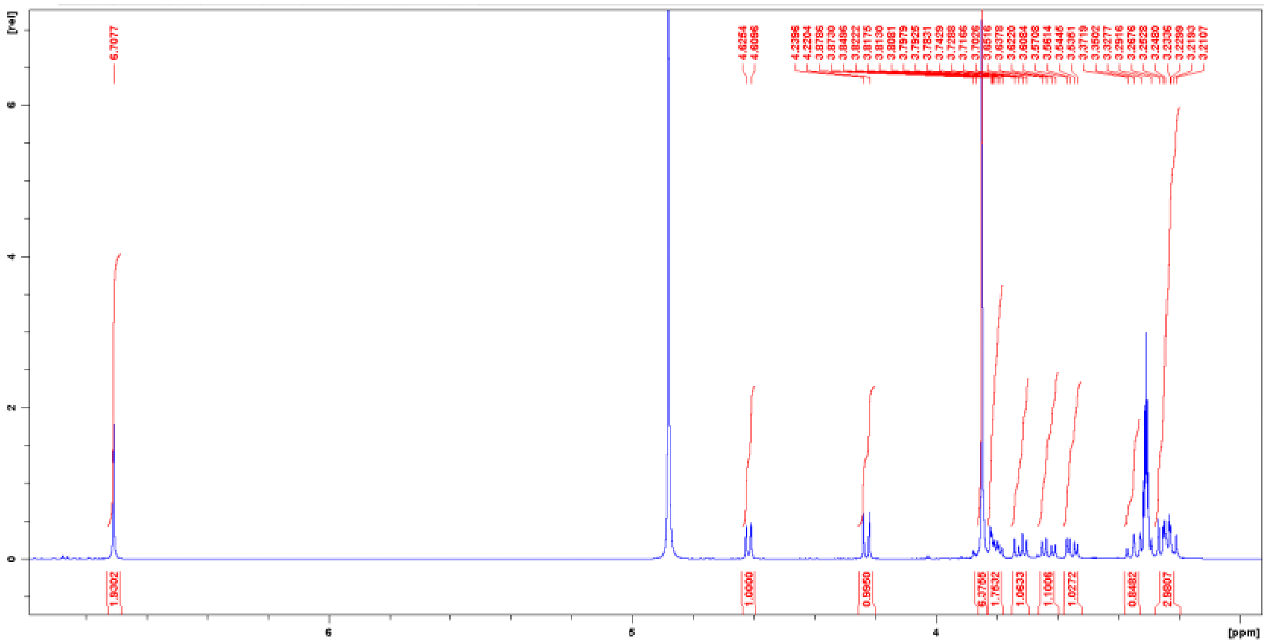

Fig. (S2). The 1H NMR spectrum of compound 5.

Table S1. Primer sequences of the melanin-related genes.

Genes	Primer sequences (5'-3')
gadph	F-ATCATCTCTGCCCCAAGTGC<br>R-AGTCAGTGGACACAACCTGG
hsp90aa1.1	F-GAGACGTTTGCGTTTCAGGCT<br>R-GGGTTTGTCTTTGTCTTCGCC
hrasa	F-CCAGGCAAGGTGTGGAAGAT<br>R-CCACTCTCGTCTGGTGGATT
pik3ri	F-TACTGTCGGGAAACGAGCAC<br>R-AGCAAGTTGTAAGGGTCCCG
mitfa	F-AGACCTGATGGCTTTCCAGT<br>R-TGTTTGGGCTGTCGTAGACC
tyr	F-CCCGAGTCAGAAGTCCTCCA<br>R-ATCAGCCCACACATTCCCAG
tyrp1	F-CTCAGTATCCTCACGACGGC<br>R-ATCTACACCCCCGAAGCTCT

Note: F denotes forward primer and R denotes reverse primer.

Table S2. 1H NMR spectroscopic data of Compound 5 and compared with the literature (δ in ppm, J in Hz).

NO.	Obs. (CD3OD, 400 MHz)	Ref. (CD3OD, 500 MHz)
2,6	6.71 (2H, s)	6.70 (2H, s)
1'	4.62 (1H, d, J= 6.3)	4.61 (1H, d, J= 6.4)
2'	3.81 (1H, m)	3.81 (1H, m)

3'a	3.72 (1H, dd, J= 10.5, 5.6)	3.71 (1H, dd, J= 10.4, 3.1)
3'b	3.55 (1H, dd, J= 10.5, 3.8)	3.55 (1H, dd, J= 10.7, 3.6)
1"	4.23 (1H, d, J= 7.7)	4.22 (1H, d, J= 7.6)
2"-5"	3.21-3.34 (4H, m)	3.21-3.34 (4H, m)
6"a	3.81 (1H, m)	3.81 (1H, m)
6"b	3.63 (1H, dd, J= 11.8, 5.5)	3.62 (1H, dd, J= 11.9,5.5)
-OCH3	3.85 (6H, s)	3.84 (6H, s)

Table S3. Target proteins with potentially critical roles in compound 5 interaction with melanogenesis.

NO.	UniProt ID	Gene Abbreviation	Degree
1	P07900	HSP90AA1	11
2	P01112	HRAS	10
3	P27986	PIK3R1	8
4	O00206	TLR4	6
5	Q14145	KEAP1	5
6	Q9NS75	CYSLTR2	5
7	Q16236	NFE2L2	4
8	P09619	PDGFRB	4
9	P16234	PDGFRA	4
10	Q14289	PTK2B	4

Table S4. Binding energies of compound 5 and its isoforms to core target proteins.

Binding Energy（Kcal/mol）	Compound 2	Compound 5	Compound 7a	Compound 7b
HSP90AA1	28.61	16.47	-6.25	-7.55
PIK3R1	652,974,919.70	51,397,452.41	-	-
HRAS	1.23	8.33	22.53	18.42
